# Sequencing asymmetry: the case for caution before first-line pirtobrutinib in chronic lymphocytic leukemia

**DOI:** 10.1007/s00277-026-07264-x

**Published:** 2026-09-01

**Authors:** Boyi Chen, Shenxian Qian

**Affiliations:** 1 The Fourth Clinical Medical College, Zhejiang Chinese Medical University, Hangzhou, 310053 China; 2 Department of Hematology, Hangzhou First People’s Hospital, Hangzhou, 310006 China

**Keywords:** Chronic lymphocytic leukemia, Pirtobrutinib, Bruton tyrosine kinase inhibitor, Drug resistance, Treatment sequencing

## Abstract

The phase III BRUIN CLL-313 and CLL-314 trials have established pirtobrutinib, a noncovalent Bruton tyrosine kinase (BTK) inhibitor, as a candidate for first-line therapy in chronic lymphocytic leukemia (CLL), and all-lines regulatory approval has now been granted in the European Union. We highlight an untested asymmetry: after failure of covalent BTK inhibitors, pirtobrutinib retains phase III–validated activity, whereas resistance to first-line pirtobrutinib can arise through kinase-domain mutations (e.g., A428D, L528W) that confer in vitro cross-resistance to covalent agents, the class that currently anchors relapse management. No clinical data exist on covalent BTK inhibitors after pirtobrutinib failure. We argue that, until reverse-sequence data emerge, a cautious approach that retains the clinically validated covalent-first sequence is reasonable for patients expected to need multiple lines of therapy, and we suggest mutation testing at progression, registry tracking of post-pirtobrutinib outcomes, and explicit discussion of sequencing in forthcoming guideline updates.

To the Editor,

The treatment of chronic lymphocytic leukemia (CLL) is approaching a regulatory inflection point. In December 2025, the phase III BRUIN CLL-313 and CLL-314 trials were published back-to-back: the noncovalent Bruton tyrosine kinase inhibitor (ncBTKi) pirtobrutinib reduced the risk of progression or death by 80% versus bendamustine–rituximab in untreated patients (hazard ratio [HR] 0.199; 24-month progression-free survival [PFS] 93.4%) [1], and was non-inferior to ibrutinib head-to-head, with approximately one-sixth the incidence of atrial fibrillation (2.4% vs. 13.5%) and a strong PFS signal in the treatment-naïve subgroup (HR 0.24) [2]. In an accompanying editorial, Davids argues these trials “open the door” for noncovalent BTK inhibition as initial therapy [3]. Regulators are following: after a positive CHMP opinion in June 2026, the European Commission approved pirtobrutinib for adults with CLL across all lines of therapy in August 2026 [4], and an FDA all-population decision is anticipated in the second half of 2026. As this transition unfolds, we flag an asymmetry neither trial was designed to address: the order in which BTK inhibitor classes are given matters, and only one order is currently supported by randomized evidence.

The covalent-first sequence rests on randomized evidence. After covalent BTK inhibitor (cBTKi) failure, most often driven by BTK C481S, which blocks covalent but not noncovalent binding, pirtobrutinib retains clear activity: a 73.3% response rate and median PFS of 19.6 months in the BRUIN phase 1/2 study, irrespective of C481 status [5], and a phase III PFS advantage over idelalisib–rituximab or bendamustine–rituximab in BRUIN CLL-321 (HR 0.54; median 14.0 vs. 8.7 months) [6]. A patient who starts on a cBTKi therefore keeps a rational, evidence-backed exit ramp.

No comparable data exist for the reverse sequence, and the resistance mechanism gives reason for concern. Wang and colleagues showed that acquired resistance to pirtobrutinib emerges not through C481 but through a distinct set of kinase-domain mutations (V416L, A428D, M437R, T474I, L528W), of which A428D and L528W conferred in vitro cross-resistance to ibrutinib and prevented multiple covalent BTK inhibitors from inhibiting mutant BTK [7]. Clinically, a patient progressing on first-line pirtobrutinib may harbor clones that have compromised not one drug but much of the covalent class, the class that currently anchors relapse management. No patient in the Wang series acquired a new C481 mutation on pirtobrutinib; the two classes select largely distinct mutations, and those selected by the ncBTKi can reduce covalent-agent sensitivity. As Mato et al. noted, “no information is currently available on the efficacy of covalent BTK inhibitors in patients who receive pirtobrutinib first and in whom resistance develops” [5]. Until sequencing data exist, the order supported by randomized salvage evidence may be the more prudent choice, particularly for patients who are likely to need multiple lines of therapy.

Davids reasonably counters that many older, comorbid patients may need only one or two lines of therapy, making downstream resistance “less salient” [3]. For that subgroup we concur: first-line pirtobrutinib is defensible where one line is the realistic horizon. But CLL is not uniformly a disease of octogenarians. Younger patients, and those with unmutated IGHV or TP53 aberration (patients with del(17p) were excluded from CLL-313), can expect disease courses of ten to twenty years and multiple lines of therapy; for them, sequencing is the central strategic problem. Nor does toxicity justify skipping the class; the “ibrutinib-era” cardiovascular argument has been overtaken by second-generation cBTKis. Zanubrutinib more than halved atrial fibrillation versus ibrutinib in ALPINE (5.2% vs. 13.3%) while improving PFS [8], and acalabrutinib reduced atrial fibrillation in ELEVATE-RR (9.4% vs. 16.0%) with non-inferior PFS [9]. Covalent-first also leaves later options intact: beyond pirtobrutinib after cBTKi failure [5, 6], the pirtobrutinib–venetoclax–rituximab triplet has outperformed venetoclax–rituximab in previously treated disease (BRUIN CLL-322, HR 0.547) [10]. Frontline pirtobrutinib therefore forgoes, at least at present, the only BTK-inhibitor class with phase III–validated salvage value.

Two qualifications are in order. First, the cross-resistance evidence is preclinical (in vitro IC50 and binding studies), and every patient in the Wang series who developed a non-C481 mutation had prior ibrutinib exposure; whether frontline pirtobrutinib selects the same mutational spectrum is unknown [7]. We therefore believe that the possibility that first-line pirtobrutinib may compromise later covalent therapy has not been excluded, and that it is most relevant for patients with long expected disease courses. Cross-resistance is also not strictly one-directional, since mutations such as L528W have been reported, less frequently, after covalent BTK inhibitor therapy [7]. These uncertainties argue for caution and surveillance, not regulatory delay. Second, BTK degraders are not yet a safety net. They degrade kinase-impaired mutants such as L528W [11], and early-phase studies of BGB-16,673 and bexobrutideg report rapid, durable responses; but the evidence remains abstract-level, pivotal trials are still accruing, and A428D can itself confer degrader resistance [12]. Degrader efficacy in this setting awaits pivotal-trial confirmation.

We suggest the following. (1) Frontline ncBTKi trials should mandate BTK and PLCG2 mutation testing at progression, so the field learns which mutations first-line pirtobrutinib actually selects. (2) Real-world registries should track responses to covalent agents after pirtobrutinib failure; this is the single most urgent evidence gap. (3) Routine practice should incorporate BTK/PLCG2 testing at progression on any BTK inhibitor. (4) We urge that sequencing be explicitly discussed in forthcoming guideline updates and that post-pirtobrutinib outcomes be systematically collected while reverse-sequence data remain absent.

The BRUIN program has given CLL a highly effective drug, and its first-line efficacy is not in doubt. What remains unknown is what early use may cost at relapse; until prospective sequencing data emerge, that uncertainty argues for an individualized, sequencing-aware choice rather than a wholesale shift of the default.

## Data Availability

No datasets were generated or analysed during the current study.

## Declarations

During the preparation of this work, AI-based tools were used for literature retrieval only; the authors reviewed and verified all content and take full responsibility for the integrity of the manuscript.
